# Androgen receptor phosphorylation status at serine 578 predicts poor outcome in prostate cancer patients

**DOI:** 10.18632/oncotarget.13608

**Published:** 2016-11-25

**Authors:** SC Patek, JM Willder, JS Heng, B Taylor, PG Horgan, HY Leung, MA Underwood, J Edwards

**Affiliations:** ^1^ Institute of Cancer Sciences, Wolfson Wohl Cancer Research Centre, University of Glasgow, Glasgow G12 8QQ, UK; ^2^ Academic Department of Surgery, School of Medicine, University of Glasgow, Walton Building, Glasgow Royal Infirmary, Glasgow, G4 0SF, UK; ^3^ Beatson Institute of Cancer Research, Glasgow G61 1BD, UK; ^4^ Department of Urology, Queen Elizabeth University Hospital, Glasgow G31 2ER, UK

**Keywords:** androgen receptor, biomarker, phosphorylation, prostate cancer, protein kinase C

## Abstract

**Purpose:**

Prostate cancer growth is dependent upon androgen receptor (AR) activation, regulated via phosphorylation. Protein kinase C (PKC) is one kinase that can mediate AR phosphorylation. This study aimed to establish if AR phosphorylation by PKC is of prognostic significance.

**Methods:**

Immunohistochemistry for AR, AR phosphorylated at Ser-81 (pAR^S81^), AR phosphorylated at Ser-578 (pAR^S578^), PKC and phosphorylated PKC (pPKC) was performed on 90 hormone-naïve prostate cancer specimens. Protein expression was quantified using the weighted histoscore method and examined with regard to clinico-pathological factors and outcome measures; time to biochemical relapse, survival from biochemical relapse and disease-specific survival.

**Results:**

Nuclear PKC expression strongly correlated with nuclear pAR^S578^ (c.c. 0.469, p=0.001) and cytoplasmic pAR^S578^ (c.c. 0.426 p=0.002). High cytoplasmic and nuclear pAR^S578^ were associated with disease-specific survival (p<0.001 and p=0.036 respectively). High nuclear PKC was associated with lower disease-specific survival when combined with high pAR^S578^ in the cytoplasm (p=0.001) and nucleus (p=0.038). Combined high total pAR^S81^ and total pAR^S578^ was associated with decreased disease-specific survival (p=0.005)

**Conclusions:**

pAR^S578^ expression is associated with poor outcome and is a potential independent prognostic marker in hormone-naïve prostate cancer. Furthermore, PKC driven AR phosphorylation may promote prostate cancer progression and provide a novel therapeutic target.

## BACKGROUND

Over the last 10 years we have observed increasing incidence and decreasing mortality trends in prostate cancer. Incidence-mortality ratios were approximately 2:1 in Western Europe prior to the introduction of PSA testing. This ratio has now increased to over 7:1, illustrating the level of over-diagnosis [1]. Many patients have indolent tumours that, before PSA testing, would not have been clinically apparent in their lifetime. Such overdiagnosis often results in overtreatment. Treatment of prostate cancer with radiation, surgery or hormone therapy is costly and even surveillance strategies are expensive. The diagnosis, treatment and 5 year follow-up cost of prostate cancer in the UK was estimated at £136, 278, 237 in 2010 [2]. It is estimated that in 2030 prostate cancer will be the most common cancer in men, with rates expected to increase by 69% compared to the number of new cases in 2007 [3]. If the predicted exponential rise in prostate cancer incidence and prevalence materialises, the cost of treatment will be unsustainable to the UK economy. Therefore the main challenge for prostate cancer research and clinical care is the quandary of how to continue driving mortality rates downward while minimising over-treatment.

Although the underlying mechanisms driving prostate carcinogenesis remain elusive, it is widely accepted that prostate cancer cell growth and survival is exquisitely dependent upon activation of the androgen receptor (AR) by androgens. This explains the high response rate of prostate cancer patients to androgen deprivation therapy (ADT). ADT reduces the level of circulating androgens and therefore levels in the prostate cancer cells resulting in AR not being activated, causing cell cycle arrest and apoptosis [4]. Furthermore, the renewed expression of serum PSA levels and AR expression in castrate-resistant disease is evidence that, even in advanced disease, prostate cancer cells remain almost exclusively dependent on the AR [5, 6]. The AR and alterations affecting its functional status are therefore likely to play an important role in the development and progression of prostate cancer.

Post-translational modification of the AR occurs at 23 known sites by phosphorylation, acetylation, SUMOylation, methylation and ubiquitination [7, 8]. Phosphorylation of the AR at serine residues is thought to inhibit proteolytic degradation and stabilize AR homo-dimers, thereby enhancing its activity [9]. AR phosphorylation may also influence transactivation of the AR since AR transcriptional activity correlates strongly with phosphorylation of specific serine residues [9]. Each of the major AR domains contains at least one serine phosphorylation site. The majority of these sites are located in the N-terminal domain (NTD), which is important for AR transactivation [8]. The hinge region contains one serine phosphorylation site, Ser-650, which regulates nuclear localization, DNA binding, and coactivator recruitment [8, 10, 11]. AR Ser-578 is located within the DNA binding domain. Protein kinase C (PKC) is the kinase predicted to be responsible for phosphorylation of AR Ser-578 [12]. Phosphorylation via PKC at this site has been linked to nuclear-cytoplasmic shuttling, DNA binding and the modulation of other functional phosphorylation sites on the AR [13]. Site directed mutagenesis of Ser-578 on the AR in castrate-resistant prostate cancer cell lines demonstrated that PKC-dependent phosphorylation was reduced on average by 50% when compared to wild type cells [13]. In addition ligands such as epidermal growth factor (EGF) have been shown to increase AR transcriptional activity and cell growth via PKC dependent AR phosphorylation at serine site 578 [13]. It is therefore plausible that alterations in AR phosphorylation, in particular at Ser-578 by PKC, may drive prostate carcinogenesis. However, few studies have explored the significance of AR phosphorylation at this site in prostate cancer in the clinical setting.

In the current study we aim to determine whether AR phosphorylation at Ser-578 is associated with clinico-pathological parameters and outcome in a cohort of hormone-naïve prostate cancer patients and if AR phosphorylation at Ser-578 correlates with PKC expression. It is hypothesised that AR phosphorylation at this site may be associated with disease progression and therefore may provide a biomarker to inform treatment decision-making.

## RESULTS

### Patient characteristics

Analysis was based on 90 hormone-naïve prostate cancer patients. Patient characteristics recorded include age, Gleason score at diagnosis, PSA at diagnosis, presence of tissue lymphovascular invasion and PSA at recurrence (Table 1). Twenty-three patients had metastases to local lymph nodes (3), bone (13) and at both sites (7). Forty-seven patients had biochemical relapse (median time to biochemical relapse 2.7yr, interquartile range 1.5–3.8). Twenty-four patients were alive at time of analysis, median follow-up 11.7yr (interquartile range 9.9–14.0). Forty-six died from prostate cancer (median time to death 4yr, interquartile range 1.9–7.2) and twenty deaths were attributed to inter-current disease (median time to death of 4.1yr, interquartile range 0.9–5.5). Table 2 shows associations with clinico-pathological parameters and clinical outcome measures.

**Table 1:** Clinico-pathological characteristics of cohort

	Patients, n (%)
**Age (<70/>70)**	34 (37.0)/56 (60.9)
**Gleason (<7/7/>7)**	24 (26.1)/ 25 (27.2)/ 28 (30.4)
**PSA at diagnosis (<10/10/>10)**	19 (20.7)/ 14 (15.2)/ 36 (39.1)
**Lymphovascular invasion (no/yes)**	84 (91.3)/ 6 (6.5)
**PSA at recurrence (<10/10/>10)**	38 (41.3)/ 1 (1.1)/ 10 (10.9)
**Ki67 (**≤**median/>median)**	46 (50)/ 39 (42.4)

Number of patients with missing data is not displayed. Values that do not give a sum of 100% is due to data being unavailable.

**Table 2:** Relationship between clinico-pathological parameters and clinical outcome measures

	Univariate analysis		
**Clinico-pathological characteristic**	**Time to biochemical relapseP value, Hazard Ratio (95% CI)**	**Survival from biochemical relapseP value, Hazard Ratio (95% CI)**	**Disease-specific survivalP value, Hazard Ratio (95% CI)**
**Age (<70/>70)**	0.260, 1.40, (0.8-2.5)	0.385, 1.44, (0.6-3.3)	**0.020, 2.11, (1.1-4.0)**
**Gleason (<7/7/>7)**	**0.004, 1.94, (1.3-2.9)**	0.060, 1.48, (0.8-2.6)	**0.007, 1.91, (1.3-2.9)**
**PSA at diagnosis (<10/10/>10)**	**0.002, 1.96, (1.3-2.9)**	0.078, 1.46, (0.8-2.7)	**0.001, 2.04, (1.3-3.3)**
**Lymphovascular invasion (no/yes)**	**0.001, 4.6, (1.7-11.6)**	0.612, 1.32, (0.5-3.9)	0.114, 2.09, (0.8-5.3)
**PSA at recurrence (<10/10/>10)**		**<0.001, 5.86, (2.8-12.2)**	**<0.001, 2.82, (1.9-4.2)**
**Metastases at any time (no/yes)**	**0.001, 3.65, (1.7-8.0)**	**0.008, 4.86, (1.4-17.4)**	**<0.001, 5.0, (2.0-12.4)**
**Ki67 (≤median vs >median)**	0.796, 1.08, (0.6-2.0)	**0.185, 1.68, (0.8-3.6)**	**0.006, 2.28, (1.2-4.2)**

The clinical variables were grouped and analysed by Kaplan Meier methods with reference to clinical outcome measures as shown. Patients were considered to have biochemical relapse dependent on treatment; radical prostatectomy serum PSA >0.2ng/ml, radical radiotherapy serum PSA of 2.0ng/ml above the post treatment nadir level, hormone treatment 2-3 consecutive rises in serum PSA levels above the nadir obtained at intervals of >2 weeks [28, 29]. Numbers in bold denote significant associations with p value <0.05.

### Protein expression analysis

**Figure 1:**
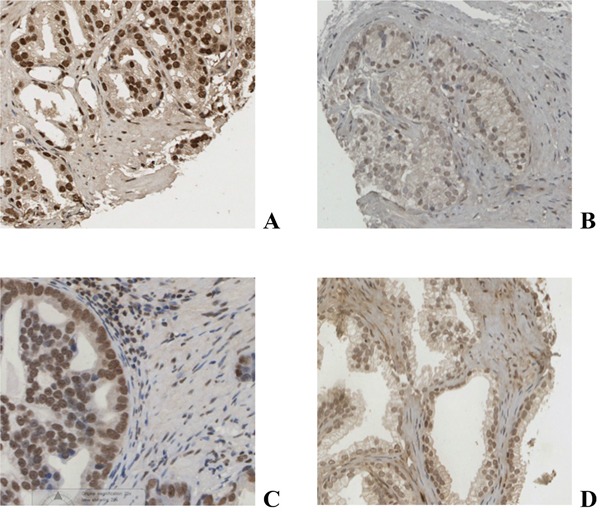
Immunohistochemical staining of prostate cancer tissue for AR A., pAR^S578^ B. PKC C. and phosphoPKC D

In addition, any tumour exhibiting negative staining for pAR^S578^ was demonstrated to have positive staining for other AR phosphorylation sites confirming that the negative expression was a true negative and not due to phospho-proteins being degraded in that particular sample. Expression of all proteins was observed at varying levels in the cytoplasm and nucleus of both stromal and epithelial cells (Figure 1). Protein expression was found to be heterogeneous throughout and less intense in the stromal cells. There was presence of prostatic intraepithelial neoplasia (PIN) and benign tissue, adjacent to the neoplastic tissue, in some of the TMA cores. Expression of proteins in the interspersed PIN and benign tissue and the normal prostate control core was heterogeneous and less intense than the neoplastic tissue. Only protein expression observed in the tumour cells was recorded. All intra-class correlation coefficient (ICCC) values were >0.80. Protein expression levels were subdivided into low (≤median) and high expression (>median) for analysis (Table 3).

**Table 3:** Protein expression in patients with tissue available

	Patients, n (%)
**AR Nuclear (low/high)**	44 (47.8)/ 45 (48.9)
**AR Cytoplasmic (low/high)**	45 (48.9)/ 44 (47.8)
**pAR^S578^ Nuclear (low/high)**	30 (32.6)/ 30 (32.6)
**pAR^S578^ Cytoplasmic (low/high)**	32 (34.8)/ 28 (30.4)
**PKC Nuclear (low/high)**	30 (32.6)/ 28 (30.4)
**PKC Cytoplasmic (low/high)**	29 (31.5)/ 29 (31.5)
**pPKC Nuclear (low/high)**	41 (44.6)/ 40 (43.5)
**pPKC Cytoplasmic (low/high)**	41 (44.6)/ 40 (43.5)

The proteins of interest were grouped as ≤median or >median. Number of patients with missing data is not displayed. Values that do not give a sum of 100% is due to data being unavailable.

### Association between protein expression and clinico-pathologic outcome measures

High expression of cytoplasmic pAR^S578^ protein was associated with increased Gleason score (p=0.008, Table 4). High expression of nuclear pAR^S578^ protein was associated with increased PSA level at diagnosis (p=0.015, Table 4). High nuclear and cytoplasmic PKC protein expression was associated with increased age (p=0.032 and p=0.018 respectively, Table 4). High expression of nuclear PKC protein was associated with increased PSA level at diagnosis (p=0.009, Table 4). Expression of pPKC was not significantly associated with any clinico-pathological outcome measures.

**Table 4:** Clinico-pathological factors related to pAR^578^ and PKC expression

	Nuclear pAR^578^			Cytoplasmic pAR^578^			Nuclear PKC			Cytoplasmic PKC		
	Low expression	High expression	p-value	Low expression	High expression	p-value	Low expression	High expression	p-value	Low expression	High expression	p-value
**Age (<70/>70)**	14/16	10/20	0.296	16/16	8/20	0.94	17/13	8/20	**0.032**	17/12	8/21	**0.018**
**Gleason (<7/7/>7)**	9/10/7	8/7/12	0.324	13/9/6	4/8/13	**0.008**	10/10/7	10/3/12	0.431	10/7/8	10/6/11	0.630
**PSA at diagnosis (<10/10/>10)**	9/4/11	2/5/19	**0.015**	8/5/13	3/4/17	0.096	9/8/9	3/3/18	**0.009**	7/7/13	5/4/14	0.478
**Lymphovascular invasion (no/yes)**	27/3	27/3	1.000	30/2	24/4	0.305	27/3	26/2	0.701	26/3	27/2	0.643
**PSA at recurrence (<10/10/>10)**	13/0/3	13/0/6	0.394	15/0/2	11/0/7	0.071	12/0/6	11/0/4	0.683	12/0/5	11/0/5	0.910
**Metastases at any time (no/yes)**	11/6	8/10	0.236	12/9	7/7	0.682	9/7	11/6	0.625	13/5	7/8	0.141
**Ki67 (≤median/>median)**	17/10	18/10	0.920	16/12	19/8	0.312	17/9	16/12	0.539	17/9	16/12	0.539

Expression of pARS^578^ in the nucleus and cytoplasm was examined for significant relationships with clinical variables as shown. Protein expression was divided into high and low groups. Clinical variables were divided into groups and used for statistical analysis. When the clinical variable consisted of 2 independent groups the Mann-Whitney U test was performed, and >2 independent groups the Kruskal-Wallis test was used. Significant associations are highlighted in bold.

### Kinase mediating AR phosphorylation

**Figure 2:**
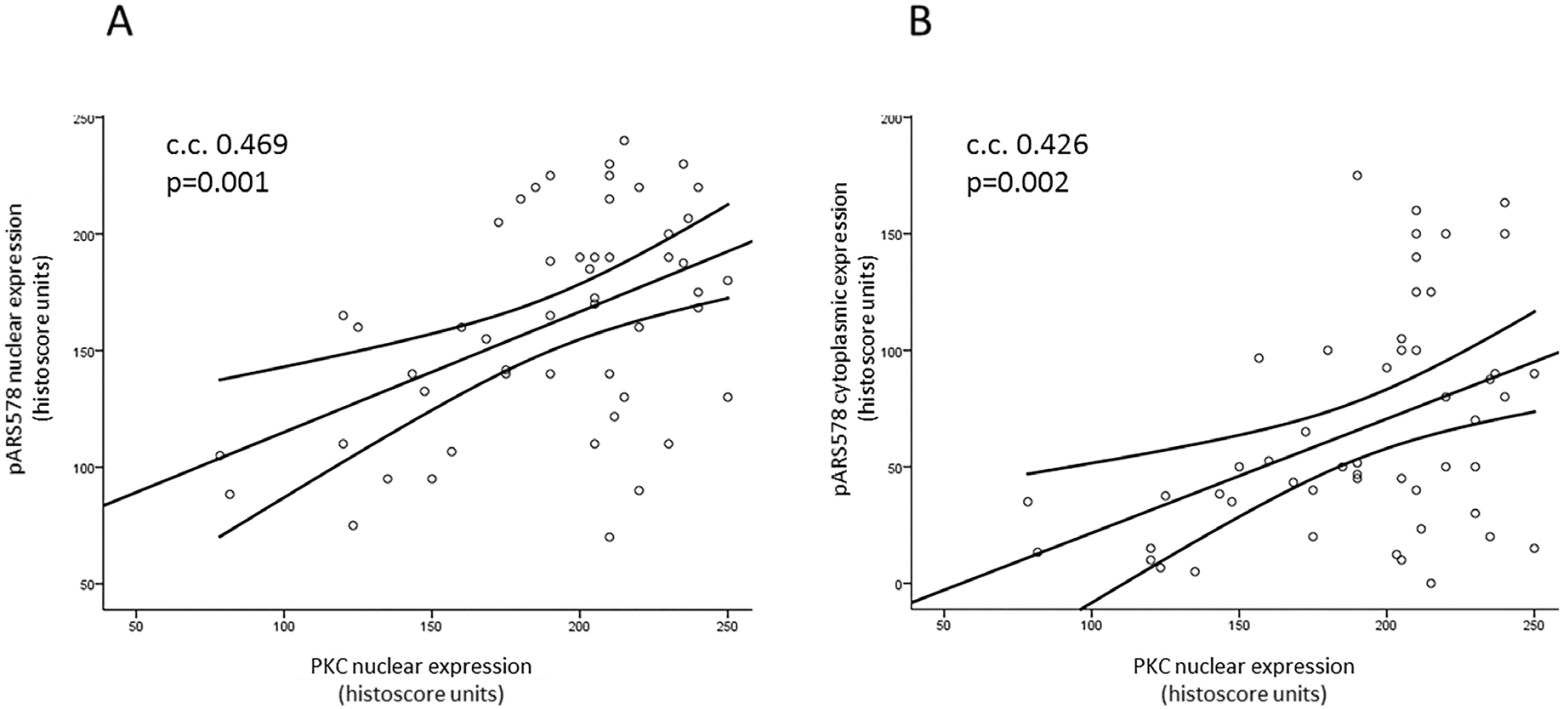
Scatter plots illustrating the correlation of PKC nuclear expression with pAR^S578^ nuclear A. and cytoplasmic B. expression Line represents best fit line with mean 95% confidence intervals. Pearson's correlation coefficient (c.c.) >0.4 and p<0.05 is considered significant.

Scansite 2.0 predicted PKC as a strong candidate mediating phosphorylation of AR at Ser-578. In the clinical specimens nuclear PKC expression was significantly associated with pAR^S578^ expression both in the cytoplasm (c.c. 0.426, p=0.002) and nucleus (c.c. 0.469, p=0.001) (Table 5, Figure 2). There was no association between pPKC expression and pAR^S578^ expression.

**Table 5:** Associations between protein expression of pAR^S578^ and kinase expression (PKC and pPKC)

	PKC		pPKC	
	NuclearP value, C.C	CytoplasmicP value, C.C	NuclearP value, C.C	CytoplasmicP value, C.C
**pAR578 Nuclear**	**0.001, 0.469**	0.044, 0.284	0.253, 0.155	0.256, 0.154
**pAR578 Cytoplasmic**	**0.002, 0.426**	0.894, 0.019	0.790, 0.036	0.649, 0.062

C.C. denotes Pearson's correlation co-efficient

Values in bold denotes associations with Pearson's correlation co-efficient > 0.4 and p value < 0.05.

### Correlation of clinical outcome with pAR^S578^ and PKC expression

High nuclear AR was associated with shorter time to biochemical relapse (proportion of patients relapsed at 5yr 78.9% vs 46.7% HR 2.84 (95% CI 1.5–5.3), p=0.001). High cytoplasmic pAR^S578^ was also associated with shorter time to biochemical relapse (proportion of patients relapsed at 5 yr 82.1% vs 51.9% HR 2.1 (95% CI 1.0-4.2), p=0.034). Interestingly, no association was observed between PKC expression and time to biochemical relapse (Table 6).

**Table 6:** Univariate analysis of AR, pAR^S578^, PKC and pPKC expression and clinical outcome measures

	Univariate analysis		
	Time to biochemical relapseP value Hazard Ratio (95% CI)	Survival from biochemical relapseP value Hazard Ratio (95% CI)	Disease-specific survivalP value Hazard Ratio (95% CI)
AR Nuclear	**0.001, 2.84, (1.5-5.3)**	0.688, 1.18, (0.5-2.7)	0.233, 1.44, (0.8-2.6)
AR Cytoplasmic	0.466, 1.23, (0.7-2.2)	0.922, 0.96, (0.4-2.1)	0.517, 1.21, (0.7-2.2)
pAR^S578^ Nuclear	0.461, 1.30, (0.6-2.6)	0.347, 1.62, (0.6-4.5)	**0.036, 2.24, (1.0-4.9)**
pAR^S578^ Cytoplasmic	**0.034, 2.1, (1.0-4.2)**	**0.034, 3.19, (1.0-9.9)**	**<0.001, 4.54, (2.0-10.4)**
PKC Nuclear	0.712, 0.88, (0.4-1.8)	0.450, 1.46, (0.5-3.9)	0.203, 1.68, (0.8-3.7)
PKC Cytoplasmic	0.938, 1.03, (0.5-2.1)	0.799, 1.14, (0.4-3.1)	0.269, 1.56, (0.7-3.5)
pPKC Nuclear	0.764, 1.10, (0.6-2.0)	0.403, 1.42, (0.6-3.2)	0.890, 1.05, (0.6-2.0)
pPKC Cytoplasmic	0.877, 0.96, (0.5-1.7)	0.647, 0.82, (0.4-1.9)	0.946, 0.98, (0.5-1.9)

Univariate analysis of AR and pAR protein expression was carried out using Kaplan Meier methods with reference to the clinical outcome measures. Patients were considered to have biochemical relapse dependent on treatment; radical prostatectomy serum PSA >0.2ng/ml, radical radiotherapy serum PSA of 2.0ng/ml above the post treatment nadir level, hormone treatment 2-3 consecutive rises in serum PSA levels above the nadir obtained at intervals of >2 weeks [28, 29]. Numbers in bold denote significant associations with p value <0.05.

Survival following disease recurrence was calculated from biochemical relapse till death or last follow-up using cancer-specific deaths. High expression of cytoplasmic pAR^S578^ was associated with less favourable survival outcomes following biochemical relapse (10 yr survival 24.3% vs 58.3% HR 3.2 (95% CI 1.0-9.9), p=0.034). No association was observed between PKC, pPKC or AR expression and survival from biochemical relapse. (Table 6)

**Figure 3:**
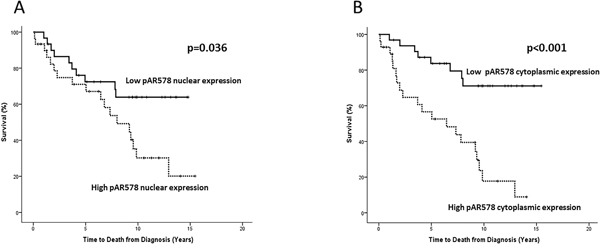
Kaplan-Meier survival plots illustrating disease-specific survival in patients with high (dashed line) and low (solid line) nuclear A. and cytoplasmic B. pARS578 expression

Disease-specific survival was calculated from diagnosis till death or last follow-up using cancer-specific deaths. High nuclear pAR^S578^ was associated with decreased disease-specific survival (10 yr survival 30.5% vs 63.8% HR 2.24 (95% CI 1.0-4.9), p=0.036) (Table 6, Figure 3). High cytoplasmic pAR^S578^ was associated with decreased disease-specific survival (10 yr survival 19.7% vs 71.3% HR 4.54 (95% CI 2.0-10.4), p<0.001) (Table 6, Figure 3). High total pAR^S578^ was associated with decreased disease-specific survival (10 yr survival 18.8% (both high) vs 75.9% (both low) HR 2.20, (95% CI 1.4-3.5) p=0.002) (Table 7). No association was observed between PKC, pPKC or AR expression and disease-specific survival.

**Table 7:** Univariate analysis of combined phosphorylated AR sites and disease-specific survival

Univariate analysis	
	Disease-specific survivalP value Hazard Ratio (95% CI)
**Total pAR^S578^**	0.002, 2.20, (1.4-3.5)
**Total pAR^S81^**	0.034, 1.78, (1.1-2.7)
**pAR^S578^ cytoplasmic + pAR^S81^ cytoplasmic**	<0.001, 2.88, (1.7-5.0)
**pAR^S578^ nuclear + pAR^S81^ nuclear**	0.011, 1.85, (1.2-2.9)
**Total pAR^S578^ + Total pAR^S81^**	0.005, 1.76, (1.3-2.4)

Univariate analysis of total and combine pAR protein expression was carried out using Kaplan Meier methods with reference to disease-specific survival.

**Figure 4:**
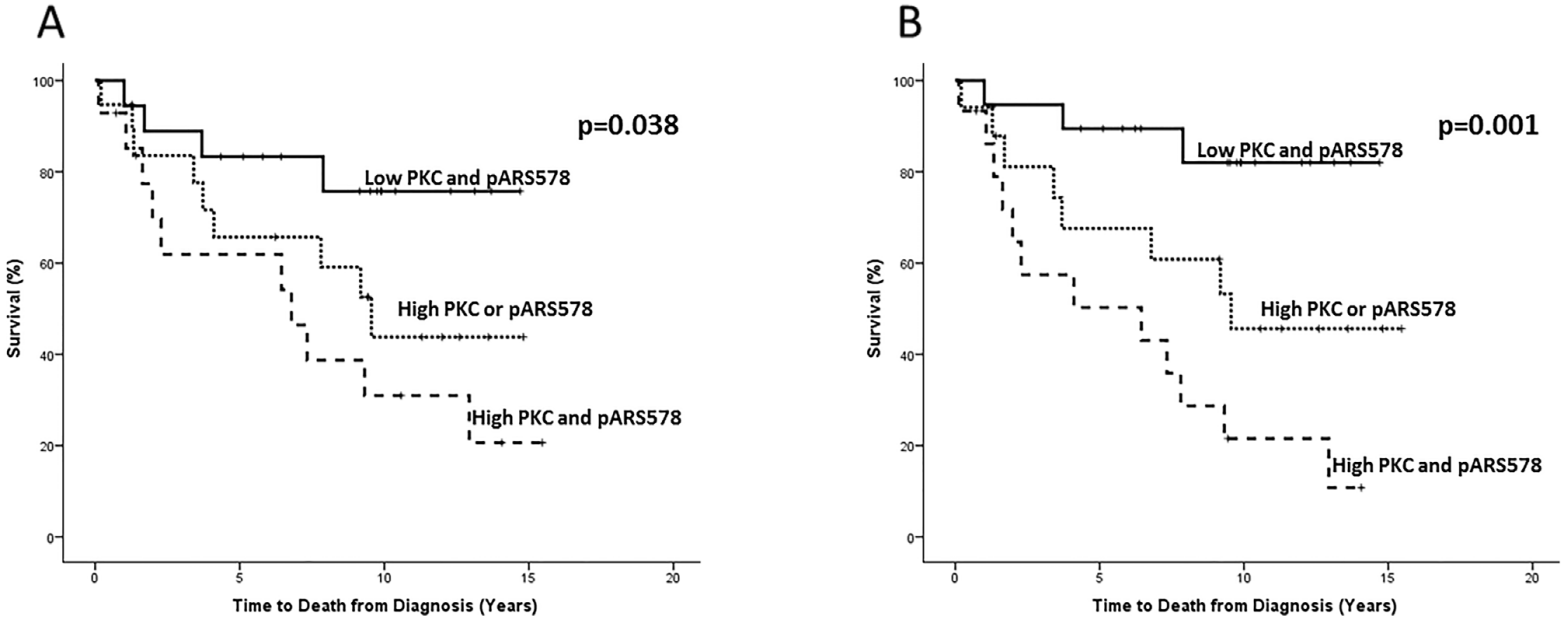
Kaplan-Meier survival plots illustrating disease-specific survival in hormone-naïve prostate cancer patients with nuclear PKC expression combined with nuclear A. and cytoplasmic B. pARS578 expression Protein expression was divided for analysis as low (≤median) and high (>median) and nuclear PKC expression was combined with pAR^S578^ expression to give the following scores; low nuclear PKC and pAR^S578^ (solid line), high nuclear PKC or pAR^S578^ (dotted line) and high nuclear PKC and pAR^S578^ (dashed line).

As nuclear PKC expression correlated strongly with pAR^S578^ both in the cytoplasm and the nucleus it was investigated as to whether a combination of the two proteins could further inform on disease outcome. Nuclear PKC expression was therefore combined with cytoplasmic and nuclear pAR^S578^ expression as follows: (i) low PKC and low pAR^S578^, (ii) high PKC or high pAR^S578^ and (iii) high PKC and high pAR^S578^ expression. High PKC and cytoplasmic pAR^S578^ expression was associated with disease-specific survival (10 yr survival 20.5% vs 46.8% vs 82% HR 2.6 (95% CI 1.5-4.6), p=0.001) (Figure 4). Similarly high PKC and nuclear pAR^S578^ expression was associated with disease-specific survival (10 yr survival 30.9% vs 44.3% vs 75.6%) HR 2.0 (95% CI 1.2-3.4), p=0.038 (Figure 4).

We have previously shown that phosphorylation of the androgen receptor at Ser-81 (pAR^S81^) is associated with decreased disease-specific survival [14]. The androgen receptor is phosphorylated at Ser-81 in response to androgens, suggesting these patients would benefit from AR targeted therapies. As it is predicted that the AR is phosphorylated by PKC at Ser-578, patients with high expression of pAR^S578^ may benefit form treatment with a PKC inhibitor. We investigated whether assessing pAR^S81^ in combination with pAR^S578^ would identify a population of patients that might benefit from dual targeted therapy (AR targeted therapies to inhibit phosphorylation at pAR^S81^ and PKC inhibitors to inhibit phosphorylation at pAR^S578^). The two phosphorylation sites were combined as follows: (i) high pAR^S81^ and high pAR^S578^, (ii) high pAR^S81^ or high pAR^S578^ and (iii) low pAR^S81^ and low pAR^S578^ expression. High nuclear pAR^S81^ and nuclear pAR^S578^ was associated with disease-specific survival (10 yr survival 20.0% vs 26.3% vs 73.2%, HR 1.85, (95% CI 1.2-2.9) p=0.011) (Table 7). High cytoplasmic pAR^S81^ and cytoplasmic pAR^S578^ was associated with disease-specific survival (10 yr survival 15.1% vs 24.4% vs 87.5%, HR 2.88, (95% CI 1.7-5.0) p=<0.001) (Table 7). Lastly, high total pAR^S81^ and total pAR^S578^ was associated with disease-specific survival (10 yr survival 0% (both high) vs 85.7% (both low), HR 1.76, (95% CI 1.3-2.4) p=0.005) (Table 7).

### Immunofluorescence

**Figure 5:**
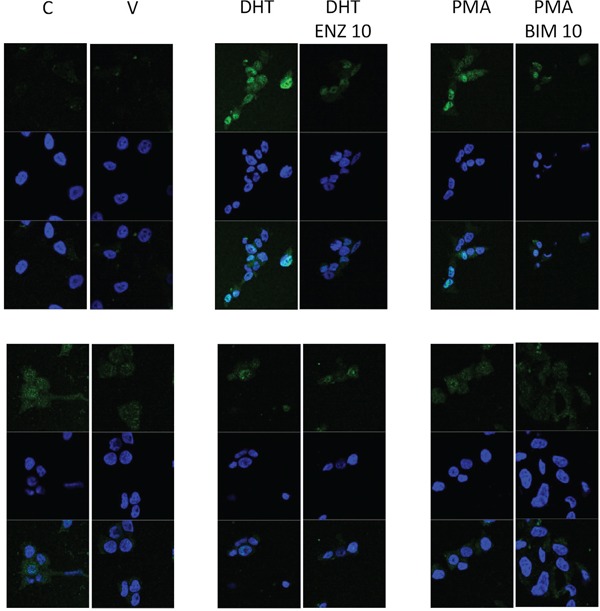
Immunofluorescent image demonstrating that nuclear translocation of AR in response to DHT or PMA can be inhibited by enzalutamide and BIM and nuclear translocation of AR578 in response to PMA can be inhibited by BIM

In LNCaP cells DHT and PMA induced AR translocation to the nucleus was inhibited by the presence of DHT and BIM respectively. DHT induced nuclear translocation of AR578 was not clearly evident in LNCaP cells suggesting that this is not regulated by DHT, AR578 nuclear translocation was observed in response to PMA and again this was inhibited by BIM, but PMA induced AR578 nuclear translocation was to a lesser extend than that observed with AR (Figure 5).

## DISCUSSION

The current study demonstrates that phosphorylation of AR at Ser-578 is strongly associated with PKC expression. In addition, the expression of pAR^S578^ in hormone-naïve prostate cancer was observed to be a negative prognostic marker associated with shorter time to biochemical relapse, decreased survival from biochemical relapse and decreased disease-specific survival. Although we were unable to demonstrate any significant association between pPKC expression and clinico-pathological outcome measures, there was a trend towards significance between high expression of both nuclear and cytoplasmic pPKC and increased Gleason score (p=0.09 and p=0.103 respectively).

In agreement with previous reports PKC expression correlated strongly with pAR^S578^ expression [13]. Previous site directed mutagenesis work in castrate-resistant cell lines has demonstrated that PKC induced phosphorylation is reduced in pAR^S578^ knock down cells [13]. The current study adds to this by suggesting that the link between PKC and AR phosphorylation is also present in hormone naïve prostate cancer tissue and therefore may have important implications in both early and late stages of the disease. Similar site directed mutagenesis studies are necessary in prostate cancer cell lines established from localised disease in order to confirm this finding.

We observed significant associations between protein expression and outcome measures for both nuclear and cytoplasmic pAR^S578^. Indeed, the presence of cytoplasmic AR is expected as the AR localizes to the cytoplasm in the absence of ligand-binding due to a ligand-regulated nuclear export signal [15, 16]. Previous mutagenesis studies investigated the effect on subcellular localization of AR in COS cells. This work demonstrated that in wild type pAR^S578^ cells AR is distributed between the nucleus and cytoplasm indicative of nuclear-cytoplasmic shuttling [13]. However in cells where the pAR^S578^ site was mutated AR expression was found exclusively in the nucleus [13]. The current study is in agreement with this work and provides further evidence for the involvement of pAR^S578^ in nuclear-cytoplasmic shuttling in neoplastic prostate tissue.

Protein kinase C is the kinase predicted to be responsible for phosphorylation at Ser-578 on the AR. We have found that whilst pAR^S578^ predicts disease-specific survival, PKC expression alone does not. When expression of pAR^S578^ was combined with PKC expression however, the two proteins together were still able to inform on disease-specific survival. No additive effect was observed in terms of significance when pAR^S578^ was combined with PKC to predict disease-specific survival, suggesting that PKC and pAR^S578^ are involved in the same pathway.

It is well established that Ser-81 is phosphorylated in response to androgen binding on the androgen receptor [8, 17]. We investigated whether there was a cumulative predictive effect in terms of disease-specific survival when two AR phosphorylation sites with two independent pathways were combined. Predictive power was increased when pAR^S81^ and pAR^S578^ expression was combined than compared to using pAR^S81^ or pAR^S578^ expression independently. These results are of great importance clinically, as it highlights a sub-population of patients who may benefit from dual targeted therapy with androgen deprivation therapy and PKC inhibitors. Unfortunately, to date, oncology clinical trials have had little success in utilising PKC inhibitors either due to poor efficacy, or significant intolerable side effects [18]. This study adds to the existing body of evidence that PKC has a role in prostate cancer, and as such encourages further work in drug development of an efficacious PKC inhibitor. Future work would consist of investigating expression of pAR^S81^ and pAR^S578^ in prostate cancer cell lines in response to treatment with androgen deprivation therapy and PKC inhibitors.

The prognostic significance of AR serine phosphorylation has been investigated previously by ourselves and others [14, 19, 20]. However, to our knowledge, this is the first *in vivo* report of the prognostic significance of AR phosphorylation at serine site 578. Previous work relating to total AR expression has demonstrated widely conflicting results. In support of its use as a negative prognostic marker in prostate cancer an investigation of 115 hormone naïve radical prostatectomy specimens demonstrated that higher tumour AR gene expression was associated with shorter time to biochemical recurrence [21]. Three further studies with a total of 788 patients demonstrated that higher AR expression was associated with a worse prognosis [22–24]. In support of this, AR gene amplification and corresponding increase in expression at the protein level has been shown in castrate-resistant tumour samples when compared to matched hormone sensitive samples from the same patient [25]. Similarly, hormone sensitive prostate cancer xenografts in castrated mice demonstrated an increase in AR gene and protein expression in addition to the acquisition of the ability of the AR to respond to anti-androgens and to aberrantly recruit coactivators to the promoters [26]. However, other work, including a large study involving a tissue microarray of 2805 prostate cancers, has shown no association of AR expression with prognosis [27]. These conflicting findings may depend on several factors such as tissue heterogeneity, timing of specimen dissection, and methods to detect AR expression including the use of different antibodies. In addition, the simple expression of AR does not reflect its function or its activity, and therefore may account for the variations reported with regards to prognostic significance. AR phosphorylation, which is known to provide molecular stability, may therefore be a marker of activation. This current study lends support to this argument as we have demonstrated that those patients who have a high level of pAR^S578^ have reduced overall survival when compared to AR expression alone.

**Figure 6:**
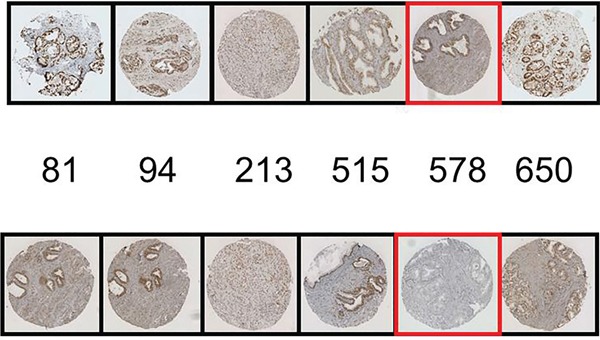
Blocking peptide experiments for pARS578 are shown The top row represents the positive control i.e. IHC conducted with each AR phosphospecific antibody as indicated. The bottom row represents antibodies to AR phosphorylation sites pre-incubated with pAR^S578^ peptide. The specific peptide utilised is boxed in red. No staining is demonstrated when the pAR^S578^ peptide is incubated with pAR^S578^antibody, whereas staining is maintained at the other phospho-AR sites.

We acknowledge the possibility of cross-reactivity in the usage of a phospho-specific antibody particularly on a protein such as AR with multiple phosphorylation sites. However the specificity of phospho-AR antibodies has been stringently tested by our group previously and they have been found to be of suitable quality (Figure 6) [14]. To follow on from both the previous and the current study we intend to validate these findings in a larger independent patient cohort.

An obvious limitation of this study is the small sample size and as such the results should be interpreted with caution and validated in a large independent cohort. However, even with low patient numbers, we have demonstrated that AR phosphorylation by PKC at serine 578 is of prognostic significance. These results are striking in particular when considered that this was a hormone-naïve cohort of patients who subsequently received a variety of treatments (surgery, radiotherapy and hormones) and that, due to small numbers, we were unable to unpick these groups. We were unable to show any significant associations between pPKC and pAR^S578^ expression, nor with any clinical outcome measures. Again, this may be due to the small sample size. We now intend to repeat this work in two further cohorts: firstly a cohort of prostate cancer patients treated with active surveillance, and secondly a larger cohort of hormone-naïve prostate cancer patients.

### conclusions

This study provides further evidence for the significance of androgen receptor signalling in the progression of prostate cancer. We have demonstrated that PKC may phosphorylate AR at serine 578 and that, in combination with current diagnostic tools, pAR^S578^ protein expression could provide a desperately needed prognostic marker to aid treatment decision-making in prostate cancer patients at diagnosis. Furthermore, PKC-driven AR phosphorylation may be a potential novel therapeutic target. This finding has the potential to reduce over-treatment of clinically insignificant disease and prevent delay in treatment of occult aggressive disease.

## MATERIALS AND METHODS

### Patients

Ninety patients with hormone-naïve prostate cancer samples available were recruited at the Glasgow Royal Infirmary between 1992 and 2002. Last date of follow up was 11/01/2012. Patients gave written consent. Clinical data included age, Gleason score, tumour lymphovascular invasion (LVI), serum PSA levels at diagnosis, biochemical recurrence, serum PSA at biochemical recurrence and presence of metastases. Patients were considered to have biochemical recurrence dependent on treatment; radical prostatectomy serum PSA >0.2ng/ml, radical radiotherapy serum PSA of 2.0ng/ml above the post treatment nadir level, hormone treatment 2-3 consecutive rises in serum PSA levels above the nadir obtained at intervals of >2 weeks [28, 29]. Study end points were biochemical relapse, survival from biochemical relapse and disease-specific survival. West of Scotland Research Ethics Committee approved the study (reference: 05/S0704/94).

### Identification of Kinases mediating AR phosphorylation

Scansite 2.0 was utilised to predict which sites on the AR would be phosphorylated by PKC [12]. The search was conducted using the protein ID “ANDR_HUMAN” (Accession number: P10275).

### Tissue microarray (TMA) construction

Three 0.6mm^2^ cores of prostate cancer tissue, identified by a uropathologist, were removed from formalin-fixed paraffin-embedded blocks. Recipient array blocks were constructed in triplicate. Control cores of normal prostate, colon, breast, pancreas, tonsil, kidney, liver and lung tissue were included in each TMA.

### Immunohistochemistry (IHC)

AR expression, pAR^S81^ expression and proliferation index (Ki67) was already available for this cohort [14]. IHC was conducted in triplicate on aforementioned TMAs for the following proteins pAR^S578^, PKC, and pPKC. Antibody to pAR^S578^ was not commercially available and so was synthesized for this project by Covalab, Villeurbanne, France. In brief, host animals were immunized with conjugated phosphorylated peptide for the protein sequence HYGALTCG[Sp]CKVF. Animals were serially bled and following the final bleed phospho-specific antibodies were selected by affinity purification. Two affinity columns were used; the first column coupled with the non-phosphorylated peptide and the second column with the phosphorylated peptide. The serum was passed through the first column and the non-retaining elute kept and used for the second purification. The elute was purified on the second column in order to remove antibodies which might recognise the un-phosphorylated peptide. ELISA tests were performed to ensure that the recovered antibody was specific for phosphorylation of AR specifically at Ser-578.

TMAs were dewaxed in xylene and rehydrated through graded alcohol. Antigen retrieval for PKC and pPKC was performed using heat treatment under pressure in citrate buffer pH6, 5min. Antigen retrieval for pAR^S578^ was performed using heat treatment under pressure in Tris-EDTA buffer (5mM Trizma Base, 1mM EDTA, pH8), 5min. Sections were cooled in buffer for 20min before washing in 3% H_2_0_2_. Sections were blocked using 5% horse serum in Tris-buffered saline (TBS). Antibodies for PKC (#ab59363, Abcam, UK), pPKC (SC -11760, Santa Cruz, USA) and pAR^S578^ (Covalab, France) were incubated overnight at 4°C diluted at 1:100, 1:120 and 1:500 respectively. All antibodies were diluted in Dako antibody diluent (Dako UK Ltd.). Bound antibody complex was visualized using EnVision plus kit (#K5007, Dako UK Ltd.) followed by 3,3-diaminobenzidine tetrahydrochloride (DAB, Dako UK Ltd.). Nuclei were counterstained with haematoxylin and Scots Tap Water Substitute. Finally, sections were dehydrated through graded alcohol and xylene and mounted with Di-N-ButylePhthalate in Xylene.

### Antibody validation

Peptide competition assays were performed to confirm pAR^S578^ antibody specificity. pAR^S578^ peptide (Protein sequence ALTCG-S(pS)-CKVFFKR raised in rabbit by Eurogentec Ltd., Seraing, Belgium) was incubated at ratio 200:1 with pAR^S81^, pAR^S94^, pAR^S308^, pAR^S515^, pAR^S578^ and pAR^S650^ antibodies overnight at 4°C. IHC was then performed as described above. Peptide competition assay demonstrated that only staining in response to antibody raised to pAR^S578^ was blocked and staining to other phosphorylation sites was not altered (Figure 6).

### Scoring

Tissue staining intensity was scored by two blinded independent observers using a weighted histo-score (H-score) method [30, 31]. H-score was calculated from the formula: (0 x % cells staining negative) + (1 x % cells staining weakly positive) + (2 x % cells staining moderately positive) + (3 x % cells staining strongly positive). The mean H-score from staining conducted in triplicate was used for analysis. Signals for nuclear and cytoplasmic pAR immunoreactivity were evaluated separately.

### Statistical analysis

Statistical analysis was performed using SPSS version 19.0 for Windows (IBM). ICCCs confirmed histo-scoring consistency between observers. Pearson's rank correlation coefficients (c.c.) assessed associations between protein expression. Mann-Whitney U test or Kruskal-Wallis test assessed relationships between protein expression and clinico-pathologic characteristics. Kaplan-Meier methods, using the log-rank test, compared survival between patients according to clinico-pathologic parameters and high/low protein expression. A <0.05 significance level was used and Bonferroni correction was applied where applicable.

### Immunofluorescence

LNCaP cells were seeded at 2.5 × 10^4^ cells per well in a 6 well chamber slide. Cells were cultured in RPMI supplemented with Glutamax, penicillin streptomycin and fetal bovine serum at 37°C in 5% CO_2_ until cells had reached 70% confluency. Cells were cultured in serum-free media for 24 hours. The LNCaP cells were treated with 1 nM dihydrotestosterone (DHT) or 1 nM phorbol 12-myristate 13-acetate (PMA), a PKC activator, or 1 nM DHT and 10 nM enzalutamide (antiandrogen) or 1n M PMA and 10 nM Bisindolymaleimide 1 (BIM-1), a PKC inhibitor, for one hour. Cells were fixed with 4% PFA at 4°C for 30 minutes. Cells were washed twice with PBS for five minutes on a rocker. Cells were permeabilised by treating twice with 0.1% Triton X-100 for five minutes. Blocking solution (1.5% horse serum in 0.1% TBS-tween) was applied for thirty minutes on a rocker at room temperature. Primary antibody, diluted in blocking solution (AR, 1:200 (DAKO), pARS578, 1:100 (Covalab)) was applied for one hour at room temperature. Cells were washed three times in TBS for ten minutes. Secondary antibody, diluted in blocking buffer, (Goat anti-mouse IgG secondary Alexa-Fluor 488, 1:500 (ThermoFisher) and Goat anti-rabbit IgG secondary Alexa-Fluor 488, 1:500 (ThermoFisher) was applied to the cells treated with the respective primary antibodies for one hour in the dark at room temperature. Cells were washed three times in TBS for five minutes. Vectashield mounting medium with DAPI (Vectorlabs) was used to counterstain DNA in the nucleus. The cells were visualised using confocal microscopy. Each experiment was performed in triplicate and repeated 3 times.
